# Enhanced *Myc* Expression in Silkworm Silk Gland Promotes DNA Replication and Silk Production

**DOI:** 10.3390/insects12040361

**Published:** 2021-04-18

**Authors:** Wenliang Qian, Yan Yang, Zheng Li, Yuting Wu, Xuechuan He, Hao Li, Daojun Cheng

**Affiliations:** 1 State Key Laboratory of Silkworm Genome Biology, Biological Science Research Center, Southwest University, Chongqing 400715, China; qianwl@swu.edu.cn (W.Q.); 18404969656@163.com (Y.Y.); lzheng92@sina.com (Z.L.); wyt1995@126.com (Y.W.); hxc9595@163.com (X.H.); leolee0822@163.com (H.L.); 2 Chongqing Key Laboratory of Sericultural Science, Chongqing Engineering and Technology Research Center for Novel Silk Materials, Southwest University, Chongqing 400715, China

**Keywords:** silkworm, silk gland, *Myc* overexpression, DNA replication, silk production

## Abstract

**Simple Summary:**

Based on a transgenic approach, enhancing *Myc* expression in the silkworm posterior silk gland (PSG), which was driven by the promoter of the fibroin heavy chain (*FibH*) gene, was performed for investigating the biological functions of Myc in silk gland. Enhanced *Myc* expression elevated the cocoon size. This elevation might be resulted from the increasing of *FibH* expression and DNA content in the PSG cells by promoting the transcription of the genes that are involved in DNA replication.

**Abstract:**

Silkworm is an economically important insect that synthetizes silk proteins for silk production in silk gland, and silk gland cells undergo endoreplication during larval period. Transcription factor Myc is essential for cell growth and proliferation. Although silkworm *Myc* gene has been identified previously, its biological functions in silkworm silk gland are still largely unknown. In this study, we examined whether enhanced *Myc* expression in silk gland could facilitate cell growth and silk production. Based on a transgenic approach, *Myc* was driven by the promoter of the fibroin heavy chain (*FibH*) gene to be successfully overexpressed in posterior silk gland. Enhanced *Myc* expression in the PSG elevated *FibH* expression by about 20% compared to the control, and also increased the weight and shell rate of the cocoon shell. Further investigation confirmed that *Myc* overexpression increased nucleus size and DNA content of the PSG cells by promoting the transcription of the genes involved in DNA replication. Therefore, we conclude that enhanced *Myc* expression promotes DNA replication and silk protein expression in endoreplicating silk gland cells, which subsequently raises silk yield.

## 1. Introduction

The silkworm (*Bombyx mori*) is an economically important insect that synthesizes silk proteins for silk production in the silk gland. The silk gland comprises three parts, namely, anterior (ASG), middle (MSG), and posterior (PSG). Cell numbers in the silk gland are determined by mitosis during the late embryonic stage [1]. During the larval stage, silk gland cells stop the mitotic cell cycle and enter into endoreplication. After approximately 17–19 rounds of endoreplicating cell cycles, also called the endocycle, the DNA content in each cell can be increased by about 400,000 times, which results in a dendritic nucleus [2,3,4].

As is well known, endoreplicating cells generally undergo multiple rounds of genome DNA replication without cell mitosis or chromosome segregation, leading to a giant cell nucleus [5,6]. Numerous studies demonstrated that both the transition of mitosis-to-endocycle and oscillation of DNA replication during the endoreplication process are determined by an upregulated expression of a scaffold protein Fzr [6,7]. Previous studies in *Drosophila* salivary gland and ovary, two tissues with an endoreplicating cell cycle, reveal that blocking Fzr expression results in an arrest of DNA replication and the failure of mitotic-to-endocycle transition [8,9,10]. Notably, the initiation of DNA replication depends on the assembling of the pre-replication complex (preRC) on the origin of DNA replication [11,12]. The mini-chromosome maintenance proteins 2–7 (MCM2-7), which are identified as preRC subunits, form a hexameric complex during the G1 phase and functions as a DNA helicase to unwind genomic DNA bidirectionally during the S phase; then, they initiate DNA replication [12,13,14]. In silkworm silk gland, oncogene *Ras1(CA)*, insulin, and ecdysone have been shown to be involved in DNA replication [15,16,17]. Undoubtedly, decoding endoreplication of silk gland cells should be helpful for better understanding silk gland growth and silk protein synthesis. Actually, PSG-specific overexpression of some growth-related regulators, such as *Ras* and *Yorkie*, can elevate silk protein genes transcription and silk production by promoting endoreplication progression and increasing DNA content in the PSG cells [15,18]. On the contrary, PSG-specific knockout of the *LaminA/C* gene, which is involved in maintaining the chromatin structure, causes a decrease in DNA content, silk protein gene transcriptions, and silk production [19].

Transcription factor Myc has been primarily identified as an oncogene in mammalian tumor cells and belongs to leucine zipper transcription factor family [20]. Previous reports in animals and plants have demonstrated that Myc is involved in regulating multiple physiological processes, such as cell proliferation and differentiation [21,22], cell growth [23], and cell self-renewal [24,25]. Enhanced *Myc* expression promotes tumorigenesis, while *Myc* deletion strongly inhibits cell activity and leads to proliferative arrest [22,26]. Increasing evidence demonstrated that Myc is involved in cell-cycle progression mainly by the initiating DNA replication and G1-S phase transition [27,28]. The observation in *Drosophila* salivary gland reveals that *Myc* heterozygous mutation induces continuous segregation of mitotic cells and prevents the entrance of endoreplication progression [29].

Previous reports in silkworm have demonstrated that silencing *Myc* expression in ovary-derived BmN4 cells causes an arrest in cell-cycle progression, and Myc is also involved in ecdysteroid regulation of cell-cycle progression in wing disc [30,31]. However, the function of Myc in silkworm silk gland with endoreplicating cell cycle remains unclear. In the present study, based on a transgenic approach, we used the promoter of the PSG-specific *FibH* gene to drive *Myc* overexpression in the PSG. PSG-specific overexpression of the *Myc* gene not only increased the size and DNA content of PSG cells but also elevated the weight and shell rate of cocoon. Mechanistically, in addition to silk protein gene *FibH*, *Myc* overexpression also upregulated the transcription of the *MCM* genes that are involved in DNA replication. These data suggest that enhanced *Myc* expression in silkworm silk gland promotes DNA replication and silk production.

## 2. Materials and Methods

### 2.1. Insect Strain 

The silkworm strain D9L (non-diapause strain) was stocked in the State Key Laboratory of Silkworm Genome Biology of Southwest University and reared with fresh mulberry leaves at 25 °C using a biochemical incubator with a cycle of 12 h light/12 h dark. The non-diapause eggs that were generated from D9L adults were used for germ-line transformation. Following the co-injection of recombinant *Myc* overexpression plasmid with helper DNA, the eggs were cultured at 25 °C with a humidity of 95–100% until hatching.

### 2.2. Construction of Recombinant Plasmid

For the construction of recombinant *Myc* overexpression plasmid pBac (3×P3-EGFP-SV40; FibH-Myc-SV40) (*FibH*-*Myc*), the opening reading frame of the silkworm *Myc* gene, *FibH* promoter, and *SV40* transcription terminator with specific restriction enzyme cutting sites were cloned into the T-simple vector. Following the digesting with corresponding restriction enzymes, the DNA fragments were successively subcloned into the pBac (3×P3-EGFP-SV40) empty vector to generate the *FibH*-*Myc* recombinant plasmid, in which PiggyBac was used as the transposable element and EGFP signal in the eyes was used for positive screening. All primers used for construction of recombinant plasmid are listed in Supplementary Table S1.

### 2.3. DNA Quantification

For DNA content quantification, 10 silk glands from the early wandering stage silkworm larvae were dissected and subsequently lysed in DNA SDS lysis-phenol supplemented with proteinase K. Following the digestion with RNAase, total genomic DNA from the middle silk gland (internal control) and the posterior silk gland were purified, separately. The DNA content was quantified spectrophotometrically at OD 260 nm using an Agilent 2100 Bioanalyzer System (Agilent, Palo Alto, CA, USA).

### 2.4. Immunostaining

After washing with PBS, the silk glands from the early wandering stage silkworm larvae were fixed with 4% paraformaldehyde for 30 min and sliced with a thickness of 10 um using a freezing microtome. Then, the slices were stained with DAPI (1:1000, Life Technologies, Carlsbad, CA, USA) for 30 min. After washing with PBS for three times, the slices were mounted with coverslips and the fluorescence signals were captured using confocal microscope Zeiss LSM 880 (Carl Zeiss, Jena, Germany) with excitation wavelengths of 341 nm.

### 2.5. RNA Extraction and Quantitative Real-Time RT-PCR (RT-qPCR)

Total RNA was extracted from the MSGs and the PSGs at the third day of the last larval instar (L5D3) using Trizol reagent (Invitrogen, Carlsbad, CA, USA), as described previously [32], and used for synthesizing cDNA templates with the M-MLV Reverse Transcriptase Kit (Promega). RT-qPCR reaction systems were prepared with a SYBR Premix ExTaq Kit (Takara, Kyoto, Japan) and performed with a qTower 2.2 Real-time PCR Detection System (Analytik Jena Biometra, Jena, Germany). The silkworm ribosomal protein L3 (RpL3) gene was used as the internal control. The relative mRNA expression levels were calculated using the 2−ΔΔCT. All primers used for RT-qPCR are listed in Supplementary Table S1.

### 2.6. Statistical Methods

The values from three independent biological replicates are presented as the mean ± SE. Statistical significance (*p*-value) was calculated by spss t-test. For the significance test: * *p* < 0.05, ** *p* < 0.01, and *** *p* < 0.001.

## 3. Results

### 3.1. Construction of Transgenic Silkworm with PSG-Specific Myc Overexpression

**Figure 1:**
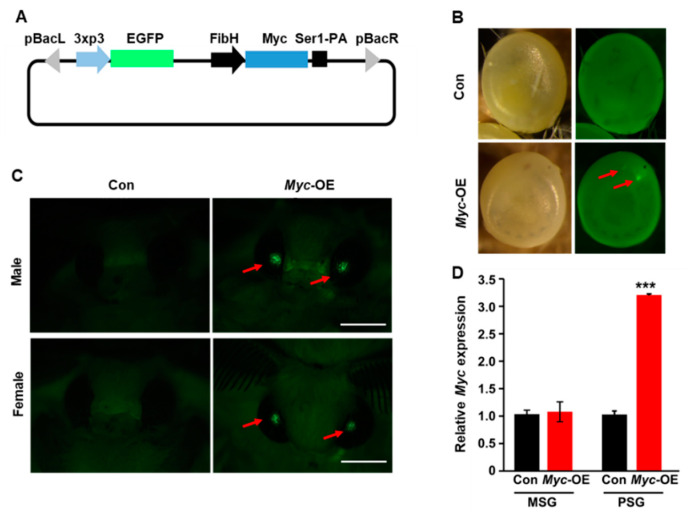
Generation of transgenic silkworm with PSG-specific *Myc* overexpression. (**A**) Schematic illustration of the vector for *Myc* overexpression driven by the *FibH* promoter. (**B**,**C**) EGFP-positive eggs (**B**) and adults (**C**) were screened in G1 generation. (**D**) *Myc* was highly expressed in the PSG of transgenic silkworm. Values are represented as means ± S.E. (error bars). For the significance test: *** *p* < 0.001 vs. the control.

To understand the roles of the *Myc* gene in both silk gland development and silk production, we first constructed transgenic silkworm with PSG-specific *Myc* overexpression. Based on the full-length sequence of silkworm *Myc* gene, we cloned the opening reading frame of the *Myc* gene and constructed recombinant *Myc* overexpression plasmid driven by *FibH* promoter, which is specifically activated in the PSG (Figure 1A). In total, 90 non-diapause D9L embryos were microinjected with the *FibH*-*Myc* recombinant plasmid, and 72 embryos were allowed to survive to develop to adults. EGFP-positive eggs in G1 generation were screened as positive transgenic strains (Figure 1B,C) and the positive rate was about 5%. Besides, the PSG of transgenic silkworm was isolated to determine whether *Myc* was specifically overexpressed in the PSG. RT-qPCR analysis confirmed that compared to the control, *Myc* was highly expressed in the PSG of transgenic silkworm (Figure 1D). These results indicate that *Myc* was specifically overexpressed in the PSG of transgenic silkworm.

### 3.2. PSG-Specific Myc Overexpression Improves Silk Yield

**Figure 2:**
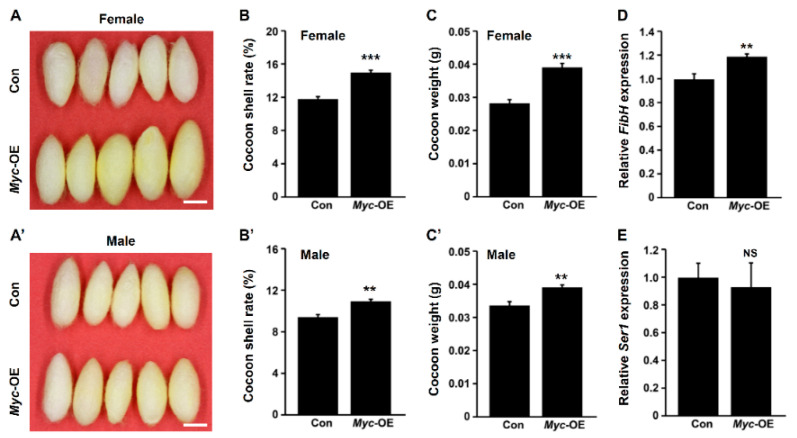
Enhanced *Myc* expression in the PSG elevates silk yield. (**A**,**A**’) Cocoon size of female (**A**) and male (**A**’) transgenic silkworms with *Myc* overexpression increased compared with control. Scale bar, 1 cm. (**B**,**C**’) The cocoon shell rates (**B**,**B**’) and cocoon weight (**C**,**C**’) were both largely increased following PSG-specific *Myc* overexpression. (**D**,**E**) *Myc* overexpression in the PSG promoted the transcription of *FibH* (**D**) but had no effect on the transcription of *Ser1* (**E**). Values are represented as means ± S.E. (error bars). For the significance test: ** *p* < 0.01, *** *p* < 0.001 vs. the control.

We next investigated the effects of PSG-specific *Myc* overexpression on silk yield. The results showed that the cocoons of female transgenic silkworm individuals with PSG-specific *Myc* overexpression were obviously bigger than that of wild-type silkworm, and the cocoons of male silkworm increased by a small amount compared to the wild-type silkworm (Figure 2A,A’). The cocoon shell rates were elevated by 25% and 22% in female and male transgenic silkworms, respectively (Figure 2B,B’). Further statistics analysis revealed that compared to the control, *Myc* overexpression led to an increase in the weight of cocoon shell (Figure 2C,C’). Moreover, the transcription of silk protein gene *FibH* increased by about 20% following *Myc* overexpression (Figure 2D), but *Myc* overexpression did not affect the transcriptions of PSG-specific fibroin light chain (*FibL*) and *P25* genes (Figure S1) as well as MSG-specific sericin 1 (*Ser1*) gene (Figure 2E). These data suggest that *Myc* overexpression in the PSG improves silk yield. 

### 3.3. PSG-specific Myc Overexpression Promotes DNA Replication 

**Figure 3:**
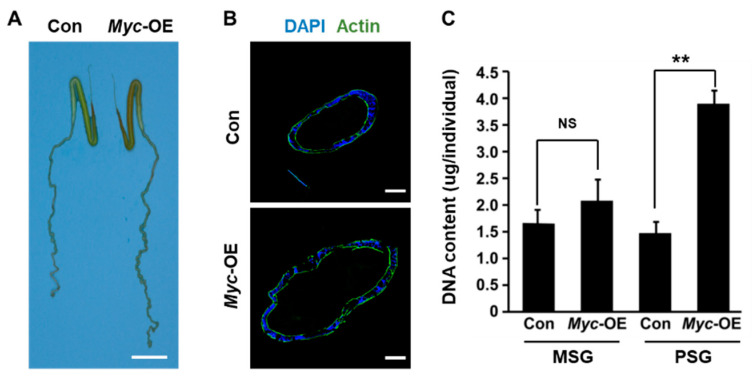
PSG-specific *Myc* overexpression increases DNA content. (**A**) *Myc* overexpression resulted in an increase in PSG size. (**B**) Immunofluorescence analysis of the nuclei size with DAPI staining. (**C**) Total DNA content in the PSG, not in the MSG, was highly increased following PSG-specific *Myc* overexpression. Values are represented as means ± S.E. (error bars). For the significance test: ** *p* < 0.01 vs. the control.

To decipher the regulatory mechanism underlying Myc-mediated improvement of silk yield, we dissected the silk gland from the early wandering stage of silkworm larvae and found that the PSG of transgenic silkworm was larger than that of the control (Figure 3A,B). However, the MSG as a negative control had no change in size following PSG-specific *Myc* overexpression (Figure 3A). Importantly, total DNA content in the PSG of transgenic silkworm was increased by nearly 2.5 times following *Myc* overexpression (Figure 3C), indicating that PSG-specific *Myc* overexpression promotes DNA replication during endoreplication in the PSG cells, which subsequently facilitates PSG growth.

### 3.4. Myc Positively Regulates the Transcription of the MCM Genes Involving in DNA Replication

**Figure 4:**
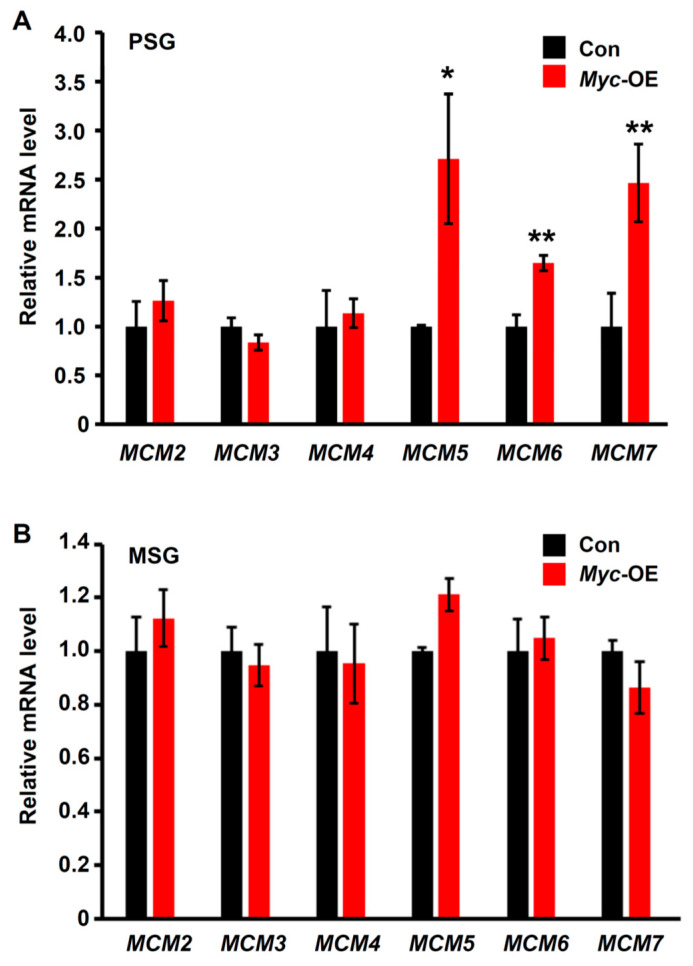
Myc positively regulates the transcription of the *MCM* genes. (**A**) RT-qPCR examination showed that PSG-specific *Myc* overexpression upregulated the transcription of the *MCM* genes in the PSG. (**B**) PSG-specific *Myc* overexpression had no effect on the transcription of the *MCM* genes in the MSG. Values are represented as means ± S.E. (error bars). For the significance test: * *p* < 0.05, ** *p* < 0.01 vs. control.

Given that enhanced *Myc* expression in the PSG cells promotes DNA replication, we further investigated whether Myc regulates the expression of the *MCM* genes, which are required for initiating DNA replication in both endocycling and mitotic cells [12]. RT-qPCR examination showed that three *MCM* genes, *MCM5*, *MCM6*, and *MCM7*, were significantly upregulated following *Myc* overexpression in the PSG (Figure 4A). As a negative control, PSG-specific *Myc* overexpression had no effect on the transcription of the *MCM* genes in the MSG (Figure 4B). Taken together, we proposed that Myc promotes the DNA replication in the endoreplicating silk gland cells by positively regulating the transcription of the *MCM* genes, which subsequently enhances the expression of silk protein gene and silk production.

## 4. Discussion

Silkworm is an economically important insect that produces silk fiber and silk proteins that are synthesized by the silk gland in which the cells undergo endoreplication. It has been demonstrated that the overexpression of *Ras1(CA)* and *Yorkie* in the silk gland improved silk yield by promoting DNA replication and increasing protein synthesis [15,18], while PSG-specific knockout of *LaminA/C* causes a decrease in DNA content, silk protein gene transcription, and silk yield [19]. DNA replication in silk gland cells can also be regulated by ecdysone and insulin [16,17]. Intriguingly, previous studies reported that ecdysone mediated DNA replication and cell proliferation in silkworm wing disc cells by positively regulated *Myc* transcription, and Myc is required for DNA replication and tissue growth in *Drosophila* endoreplicating tissues [10,29,30,33]. Accordingly, we here conducted a transgenic overexpression of the *Myc* gene in the PSG and observed that enhanced *Myc* expression promotes DNA replication and silk protein synthesis. These data indicate that Myc plays conserved roles in regulating DNA replication and protein synthesis in different types of endoreplicating cells. 

The members of the MCM family, MCM2-MCM7, interacted physically to form a hexameric complex and colocalized at assembled replication origins to initiate DNA synthesis in endoreplicating cells [12,13,14]. This hexameric helicase complex is essential for DNA replication by providing a platform for recruitment of other preRC subunits and bidirectionally unwinding genomic DNA [34,35]. Our previous study found that *Drosophila* Myc positively regulated the transcription of the *MCM6* gene by directly binding to a specific motif within its promoter during endoreplication [10]. We here observed that *Myc* overexpression in silkworm PSG can upregulate the transcription of three members of the MCM family, *MCM5*, *MCM6*, and *MCM7*, which is most likely correlated with an increase in DNA content following *Myc* overexpression. Whether Myc can directly bind to the promoter of silkworm *MCM* genes needs further investigation.

High expression of silk proteins in silkworm silk gland is required for silk production. We found that enhanced *Myc* expression in the PSG elevates both the transcription of silk protein gene *FibH* and silk production. This elevation may be associated with a *Myc* overexpression-caused increase in DNA content. In addition, the transcription of the *FibH* gene can be regulated by other silk gland-specific transcription by its direct binding to the *FibH* promoter, including basic helix-loop-helix transcription factor Sage [36], nuclear hormone receptor FTZ-F1 [37], and fibroin modulator binding protein-1 [38]. It should be necessary for elucidating whether Myc can directly regulate the transcription of silk protein genes. 

## 5. Conclusions

In conclusion, we suggest that enhanced *Myc* expression in the PSG can promote silk yield by increasing DNA content and *FibH* transcription. This provides a novel target for improving silk production in silkworm breeding.

## Data Availability

Not applicable.

## Supplementary Materials

The following are available online at https://www.mdpi.com/article/10.3390/insects12040361/s1, Table S1: The primers used in this study. Figure S1: Effect of PSG-specific Myc overexpression on the transcription of *FibL* and *P25* genes. *Myc* overexpression in the PSG had no effect on the transcription of *FibL* (A) and *P25* (B) genes
