# Supplementary material for: Enhanced Myc Expression in Silkworm Silk Gland Promotes DNA Replication and Silk Production
Source: Insects. 2021 Apr 18;12(4):361. doi: 10.3390/insects12040361 (PMC8073660; doi:10.3390/insects12040361)

Supplementary Table S1. The primers used in this study

Purpose	Primer name	Primer sequence
Construction of recombinant plasmid	*FibH* promoter	Forward: 5' CCGCTCGAGTAATTAGGTAGTGTTTAAGCTTGTTG 3'
		Reverse: 5' GGACTAGTGAGAGTTGGAACCGAACTG 3'
	Myc ORF	Forward: 5' CTAGACTAGTATGTCGCCGCCGCTAGA 3'
		Reverse: 5' CGCGGATCCTCAGTACGGGTGGTAGCGA 3'
	Ser-PA	Forward: 5' CGCGGATCCTACAACTAAACACGACTTGGAGT 3'
		Reverse: 5' CGACGCGTTTCGTCAATGTATCAGTTTTGGT 3'
RT-qPCR	*MCM2*	Forward: 5' GATGAAAATGCCGATGCC 3'
		Reverse: 5' AACTCAACATGAAAGGATGC 3'
	MCM3	Forward: 5' CAGCCGAATCCATTTACG 3'
		Reverse: 5' CTTGCCATGTCGCTATCC 3'
	MCM4	Forward: 5' TGATATAAGTCTGATGCGGGA 3'
		Reverse: 5' AGTTCAACAACGGAAGAAAGTC 3'
	MCM5	Forward: 5' GTTTTGGCTGATGGTGGT 3'
		Reverse: 5' CGAGAATTTAATGTAGTGGTGA 3'
	MCM6	Forward: 5' AGATACGAATCCAGGAAACCC 3'
		Reverse: 5' CACATCAGGCACTACAATCAG 3'
	MCM7	Forward: 5' TAGTCTTGCCCCCGAAAT 3'
		Reverse: 5' CAAAACAGCAGCAGTGAGG 3'
	FibH	Forward: 5' TCTGTGTCATCTGCTTCATCTCG 3'
		Reverse: 5' TATCCAGGACGAAGTAAGAAACAA 3'
	Ser1	Forward: 5' CACAACCGATAAGACGAG 3'
		Reverse: 5' GACGAAGTGGAGGAAGC 3'
	FibL	Forward: 5' ATACCGATTGGTCACATAACAG 3'

	Reverse: 5' GCAGATAGATGGGCGATAA 3'
P25	Forward: 5' AGCCGCTGTGGCAGTTTTG 3'
	Reverse: 5' TAGGTGGCGTTGAAGTATGG 3'

Figure S1. Effect of PSG-specific Myc overexpression on the transcription of *FibL* and *P25* genes. *Myc* overexpression in the PSG had no effect on the transcription of *FibL* (A) and *P25* (B) genes

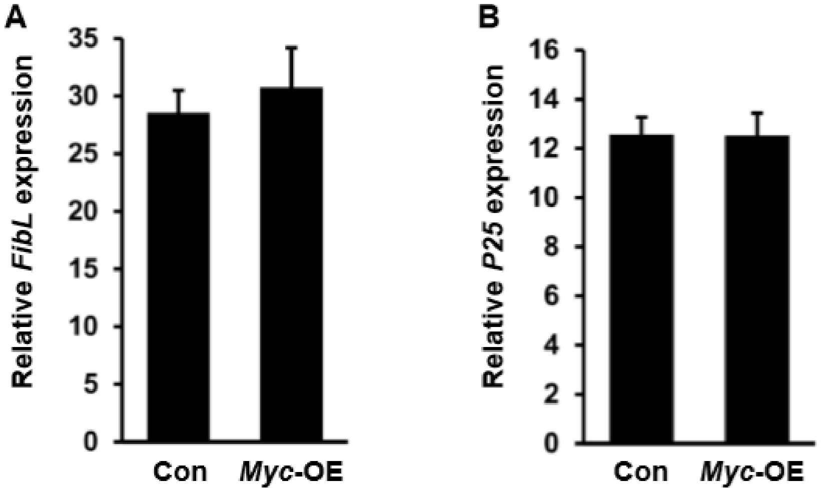

Supplement: Supplementary file 1 [file insects-12-00361-s001.zip › Supplementary Files.pdf]
